# Novel Insights into Sporotrichosis and Diabetes

**DOI:** 10.3390/jof10080527

**Published:** 2024-07-29

**Authors:** Mariana de Araujo Oliveira, Sandro Rogério de Almeida, Joilson O. Martins

**Affiliations:** 1 Laboratory of Immunoendocrinology, Department of Clinical and Toxicological Analyses, School of Pharmaceutical Sciences, University of São Paulo, São Paulo 05508-000, SP, Brazil; marianadearaujo.oliveira@usp.br; 2 Laboratory of Mycology, Department of Clinical and Toxicological Analyses, School of Pharmaceutical Sciences, University of São Paulo, São Paulo 05508-000, SP, Brazil

**Keywords:** sporotrichosis, *Sporothrix*, infection, diabetes mellitus, immunosuppression

## Abstract

Sporotrichosis is a type of zoonotic subcutaneous mycosis caused by different species of dimorphic fungus of the genus *Sporothrix*, and it is the most common form of subcutaneous mycosis in Latin America. Sporotrichosis is generally restricted to cutaneous and lymphatic tissue (i.e., localized forms), and involvement in the viscera (i.e., disseminated or disseminated cutaneous form) is uncommon, especially in the central nervous system. However, immunosuppression in individuals with diabetes mellitus can lead to the disseminated form of the disease due to a failure to eliminate the pathogen and poor infection treatment outcomes. Possible correlations between patients with diabetes and their greater susceptibility to disseminated cases of sporotrichosis include a decreased cytokine response after stimulation, increased oxidative stress, decreased chemotaxis, phagocytic activity, adhesion and rolling of neutrophils and monocytes/macrophages, and increased macrophage/monocyte and polymorphonuclear cell apoptosis. Therefore, this review highlights novel insights into diabetes and sporotrichosis by investigating how chronic inflammation affects and aggravates the infection, the possible causes of the greater susceptibility of *Sporothrix* sp. to hematogenous dissemination in immunocompromised patients, and the main alterations that this dissemination can cause.

## 1. Introduction

The incidence of zoonotic sporotrichosis, a type of subcutaneous mycosis, has increased significantly, especially in Brazil, and it is recognized as an emerging disease. With outbreaks of cases recorded in Rio de Janeiro and Itaquera—SP, addressing sporotrichosis requires an approach that involves health policies for animals and humans that aim to reduce the transmission of *Sporothrix*. In addition, it is essential to acquire more in-depth knowledge about the fungus to improve the treatments available and prevent infection.

The incidence of infections is greater in individuals with diabetes mellitus (DM) than in individuals without DM, and individuals with DM are more likely to have a complicated course of infection than individuals without DM. This phenomenon has been observed for sporotrichosis. If left untreated, it rarely leads to death in humans. However, individuals with a deficient immune system, such as those with diabetes, can develop the disseminated form of the disease, which has a high mortality rate.

The following question then arises: What pathogenic mechanisms are responsible for this high rate of infection and worsening of sporotrichosis in patients with DM? Some of the possible causes are related to the chronic hyperglycemic environment, which leads to immune dysfunction, increased microorganism adhesion to the cells of individuals with diabetes, micro- and macrovascular lesions, neuropathy, and numerous medical interventions in this group of patients [1,2].

The present review highlights novel insights into diabetes and sporotrichosis by investigating how chronic inflammation affects and aggravates infection, the possible causes of the greater susceptibility of *Sporothrix* sp. to hematogenous dissemination in immunocompromised patients, and the main alterations that this dissemination can cause.

## 2. Sporotrichosis: Etiological Agents

Sporotrichosis is a zoonotic subcutaneous mycosis caused by different species of dimorphic fungus of the genus *Sporothrix*, which is comprised of 53 species, including *Sporothrix brasiliensis*, *Sporothrix globosa*, *Sporothrix mexicana*, *Sporothrix luriei*, *Sporothrix pallida*, and *S. schenckii sensu stricto* [3,4]. *S. schenckii*, *S. brasiliensis,* and *S. globosa* are the most prevalent species [4] and are associated with human infections. The environmental clade, including *S. mexicana* and *S. pallida*, is rarely involved in human sporotrichosis but is responsible for opportunistic infections. In these situations, the infection route generally involves traumatic inoculation of the fungus present in the soil and organic matter [5,6,7,8]. Occurring primarily in tropical and subtropical regions, sporotrichosis is the most common subcutaneous mycosis in Latin America [9], and it is endemic in South America and hyperendemic in the metropolitan region of Rio de Janeiro, Brazil.

Because sporotrichosis is a dimorphic fungus, it takes two distinct forms: filamentous (in the saprophytic phase), when found in the environment or cultivated at 25 °C, and yeast-like (in the parasitic phase), when cultivated at 37 °C or inside the host [10,11]. Environmental species typically exhibit low virulence because the conversion from mycelium to yeast, which successfully occurs among clinical clade species, is deficient in environmental species and results in few yeast-like cells [7].

Experimental studies have shown that the virulence profiles vary according to the pathogen characteristics and the host immune response. However, *S. brasiliensis* stands out as the most virulent species in terms of mortality, tissue burden, and tissue damage [12,13,14]. *S. schenckii* exhibits varying levels of virulence [12,15,16], but *S. globosa*, *S. mexicana*, and *S. albicans* have shown low or no virulence in the animal models used and low invasive potential [6,12].

One of the characteristics of *S. brasiliensis* is a pronounced cerebral tropism compared to other species [12]. This phenomenon has been observed in clinical practice, and severe cases of meningeal sporotrichosis have been described in immunocompromised and immunocompetent patients [17,18,19].

In Brazil, the etiological agent of sporotrichosis is *S. brasiliensis*, which is associated with zoonotic transmission by infected cats due to the large amount of viable yeast present in skin lesions and other tissues; transmission occurs via contact with the exudate of the lesions of infected animals and traumatic inoculation in subcutaneous tissue via bites or scratches by infected animals [20,21,22,23,24], which results in atypical cases of the disease [16,25].

The healing process typically requires approximately three months of antifungal medication. Studies have reported an increase in the number of strains resistant to amphotericin B and itraconazole [26], which highlights the importance of seeking alternative therapies.

In vitro studies evaluated the antifungal drugs terbinafine, ketoconazole, and posaconazole, which are widely used to treat sporotrichosis, and *S. brasiliensis* had the best response. In contrast, these same antifungal drugs showed very low activity against *S. albicans*, *S. globosa*, and *S. mexicana* [27]. One of the factors that may be involved in the resistance to some antifungal drugs is the ability of *Sporothrix* to produce melanin, which is an important virulence factor that allows the fungus to evade host defenses and medications [7,13,28].

Although death from sporotrichosis is uncommon in untreated humans, individuals with compromised immune systems, such as those with diabetes, may develop the disseminated form of the disease [29,30].

## 3. Epidemiology of Sporotrichosis in Brazil

**Figure 1:**
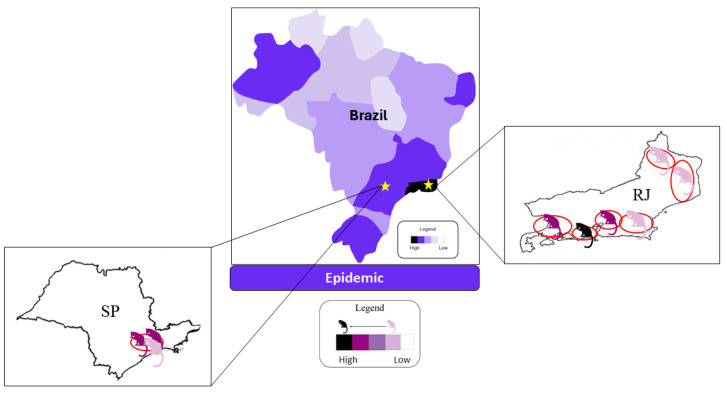
Distribution of feline sporotrichosis cases in Brazil, focusing on São Paulo and Rio de Janeiro, in recent years. The map was constructed based on case reports available in the literature showing a scenario of evident fungal infection [8,21,22,32,33,34,35,36,37,38,39,40,41,42,43,44,45,46].

As the most common subcutaneous mycosis in Latin America [7,31], zoonotic sporotrichosis was first documented in Brazil in 1955 in the state of São Paulo [8]. It is most common in southern and southeastern Brazil, with the states of São Paulo, Rio de Janeiro, and Rio Grande do Sul being the most affected (Figure 1).

Between the late 1950s and the early 1990s, zoonotic transmission was infrequent in the southeastern region of Brazil [47]. However, from 1990 onward, there was an increase in the number of feline cases in the municipality of Rio de Janeiro and in Paraná during the 2000s [42]. During this period, *S. schenckii* in the oral cavity and claws of infected cats was isolated [48].

Since 1998, cases of feline sporotrichosis have been reported in several municipalities in the state of Rio de Janeiro. In São Paulo, the predominance of cases is observed in the northern and eastern areas of the capital and the municipality of Guarulhos-SP [21,32,33,42,43].

Approximately 60 years after the first description of cases of zoonotic transmission, this issue has become a major public health challenge in Brazil, especially in the state of Rio de Janeiro. As a result of this epidemic, notification has been mandatory in the state of Rio de Janeiro since 2011 but not in other Brazilian states [36,37].

## 4. Transmission and Clinical Manifestations of Sporotrichosis: Humans and Animals

Transmission can occur in two ways: environmental (i.e., sapronotic), where the fungus present in the environment comes into contact with the skin or mucosa via injuries caused by plant matter, its byproducts, or soil, such as accidents with thorns; or animal (i.e., zoonotic), where the main source of infection for humans and other animals is the domestic cat (*Felis catus*).

Sporotrichosis manifests as the appearance of a papule or nodule at the site of traumatic inoculation after a few days. When the fungus penetrates the host’s body, it takes on a yeast-like form [33,49].

Sporotrichosis is generally restricted to cutaneous and lymphatic tissue (i.e., localized forms), but involvement with viscera (i.e., disseminated or disseminated cutaneous form) is uncommon, especially in the central nervous system (CNS) [50]. Pulmonary, bone, and genitourinary manifestations have also been described. On rare occasions, sporotrichosis can spread to other organs. Patients with neoplasms of the hematopoietic or lymphoreticular system, AIDS, diabetes, alcoholism, or intensive use of immunosuppressant substances can present with the disseminated form of the disease [50].

The lymphocutaneous form is the most frequent clinical form, and it was reported in approximately 80% of the cases. However, the first case of CNS sporotrichosis was confirmed and published in a case study in 1953. A 42-year-old man with no associated conditions developed multiple cerebral granulomas and died after two months [51]. Other cases have been reported since 1998 and are generally associated with immunosuppression [52], which demonstrates that host immunity is an important factor in the clinical manifestations of this disease.

The role of cats in transmission is amplified by feline behavior, which includes nocturnal habits for hunting and reproductive rituals, and territorial fights and disputes over females in heat. During these periods, animals with ulcerated skin lesions and the presence of yeast may cause sporotrichosis via direct contact with wounds, bites, and scratches, especially on the face [21].

## 5. Sporotrichosis: Immune Response

After inoculation with the fungus, innate immune mechanisms are activated to directly control the infection and stimulate a specific immune response involving the Th1 and Th17 subsets of T lymphocytes [53,54]. Cell-mediated innate immunity plays an important role in the control of feline sporotrichosis, based on the increased percentage of CD4+ T cells in cats with single lesions, well-organized inflammation, and a decreased fungal load. When cats have lesions with granulomas and a high fungal load, they have low CD8+ T-cell counts [35].

For sporotrichosis to develop in the host body, the conidia must undergo a dimorphic transformation when they come into contact with macrophages and become yeasts. The survival of the fungus is made possible by the low induction of a proinflammatory response and cell death induced by reactive oxygen species (ROS) [55]. This characteristic is crucial for the virulence of the fungus because the transition from hyphae to yeast results in changes to the cell wall, which exposes the antigenic components [56].

In addition, the ergosterol present in the fungus’s cell membrane reacts with the hydrogen peroxide produced by macrophages to form ergosterol peroxide, which is one of the evasion mechanisms of the fungus [57]. Previous studies have shown that melanin, which is produced from phenolic compounds, such as l-3,4-dihydroxyphenylalanine (L-DOPA) [3], confers fungal resistance against phagocytosis and protects against UV radiation, temperature increases, and reactive oxygen species [58,59]. As described by Almeida-Paes et al. (2009) [3], *S. brasiliensis* species exhibit rapid melanization and high levels of pigmentation.

**Figure 2:**
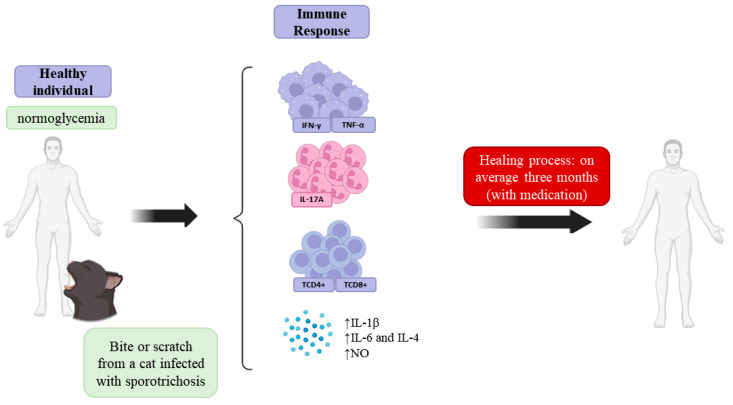
Innate, cellular, and humoral immune responses play important roles in controlling sporotrichosis. After inoculation, innate immune mechanisms are activated to control infection directly and stimulate a specific immune response involving Th1 and Th17 subsets of T lymphocytes. APCs (antigen-presenting cells) recognize and phagocytose fungi, after which immunogenic peptides are presented to T lymphocytes. TCD4+ lymphocytes, macrophages, CD8^+^ cells, and IFN-γ production are important for granuloma formation. After two months of infection, the IL-1β, IL-6, IL-4, and TNF-α levels increase. Macrophage activation occurs primarily via TNF-α, which stimulates macrophages to produce NO (nitric oxide), which is highly cytotoxic to *Sporothrix* sp.

There are many gaps in knowledge of the immune response to *Sporothrix* sp., but macrophages play crucial roles in initiating, maintaining, and resolving the inflammatory response in the host. These cells act as regulatory effectors of the immune system by recognizing, phagocytosing, and processing the etiological agent for subsequent antigen presentation. After activation, macrophages phagocytose the invading pathogens and promote the production of proinflammatory cytokines, such as IL-6, IL-4, TNF-α, and IL-1β. These cytokines stimulate the phagocytic responses and promote a release of toxic agents that mediate the immune and inflammatory responses, such as nitric oxide (NO), which is highly cytotoxic to *S. schenckii* and is released at the beginning and end of infection [60,61,62], as described in Figure 2.

Studies have shown the importance of Toll-like 2 (TLR2) and Toll-like 4 (TLR4) receptors in the innate immune response against *Sporothrix brasiliensis*. These receptors are responsible for detecting various components present in bacteria and fungi, primarily bacterial lipoproteins and tri-/diacylatolipopeptides. The absence of TLR2 and TLR4 results in impaired phagocytosis and reduced levels of TNF-α, IL-6, IL-10, and nitric oxide, which could promote a persistent infection [63,64,65].

The increase in IL-1β and IL-18 in sporotrichosis is also related to the activation of caspase-1 [54]. Th17 cells are crucial in the defense against fungi because these cells are involved in the activation and repair of epithelial barriers via the secretion of IL-17A [66].

An improvement in the environment is observed when there is a shift toward a Th2 response, although the host’s protective immunity against fungal infections seems to depend primarily on a response that is inclined toward the Th1 profile, with the activation of macrophages [53,67].

## 6. Diabetes Mellitus: Types and Epidemiology in Brazil

Diabetes mellitus (DM) is a complex syndrome that is characterized by the loss of glucose metabolism homeostasis, which leads to chronic hyperglycemia due to insufficient insulin production by the pancreas or the ineffective use of insulin produced by the body [68,69,70,71]. This loss of homeostasis causes oxidative damage and activates inflammatory signaling cascades [72,73].

In association with the high mortality and morbidity rates, which are primarily due to the greater susceptibility of individuals with DM than nondiabetic individuals to infections [74], DM may be classified into four types according to its etiopathogenesis: type 1 DM (T1DM), type 2 DM (T2DM), gestational diabetes (GD), and other specific types of diabetes [71,75,76].

Chronic hyperglycemia affects various organs, such as the brain, kidneys, heart, and eyes, and it increases the risk of various diseases caused by macro- and microvascular damage. In addition, poorly controlled glycemia causes weakness in smaller and thinner vessels, which prevents the immune system cells from reaching these sites and leads to microorganism colonization [77].

According to the International Diabetes Federation, there are approximately 537 million people with diabetes worldwide, and this number is expected to increase to 643 million by 2030 and 700 million by 2045 [78]. Brazil ranks sixth as the country with the highest incidence of patients diagnosed with diabetes in the world, with more than 16.8 million cases reported in people aged between 20 and 79 years. This number represents approximately 6.9% of the national population, with estimates suggesting that the number of cases of the disease in 2030 could reach 21.5 million Brazilians [30,78].

In 2022, approximately 8.75 million individuals were diagnosed with T1DM worldwide, with 1.9 million of these individuals living in low- and lower-middle-income countries [78]. Among the total population with T1DM, 1.52 million (17%) were younger than 20 years, 5.56 million (64%) were aged between 20 and 59 years, and 1.67 million (19.9%) were aged 60 years or older. In Brazil, 588,800 people live with T1DM [78], which places the country third among countries with the most patients diagnosed with this disease. Approximately 90% of T2DM patients in Brazil have this type of diabetes [71,78].

## 7. Immune Response of Patients with DM

To prevent the entry of pathogens, the immune system uses various physical and chemical barriers, such as natural barriers, which include the skin and mucosa, and the production of cytokines, chemokines, and reactive oxygen species (ROS). However, in individuals with DM, dysfunctions associated with inflammation result in the inability of the immune system to defend against invasive microorganisms and in increased susceptibility to infections [79,80].

Pasquel et al. [81] indicated that the chronic hyperglycemic stress present in individuals with DM increased hospital complications, length of stay, and mortality due to a greater susceptibility to autoimmune diseases and infections by microorganisms as a result of an impaired immune response [71]. In addition, hyperglycemia provides a favorable environment for fungi and bacteria to proliferate in various areas of the body.

Neely et al. [82] revealed that individuals with diabetes had a greater incidence of infections, including bacterial infections, such as osteomyelitis, pyelonephritis, cystitis, pneumonia, cellulitis, sepsis, and peritonitis, as well as fungal infections, compared to individuals without diabetes.

Some disrupted immune processes in these individuals involve reduced interactions between leukocytes and endothelial cells, reduced numbers of leukocytes in inflammatory lesions [83,84], reduced tumor necrosis factor-α (TNF-α) and interleukin-1β (IL-1β) release [85,86,87], reduced lymph node retention capacity [88], reduced mast cell degranulation and levels of inflammatory mediators, such as histamine and bradykinin [89,90], impaired phagocyte function and reduced proinflammatory mediator synthesis [84,85,86,91,92,93]. All of these factors contribute to a greater susceptibility to and worsening of secondary infections in patients with diabetes.

During an inflammatory response, leukocytes roll along the endothelium, lining the postcapillary venules, and attach to the vascular wall before migrating to tissues. Individuals with DM have leukocyte abnormalities, and one possible explanation is the negative regulation of adhesion molecules, such as intercellular adhesion molecule (ICAM)-1, which are responsible for regulating leukocyte recruitment during the inflammatory process [94,95,96].

During the host inflammatory response to infection, neutrophils play a crucial role [96]. Patients with diabetes have lower neutrophil chemotactic activity [97], impaired phagocytic and microbicidal functions (because a certain number of enzymes are insulin-dependent), decreased lysosomal enzyme release, impaired endothelium adhesion, reduced reactive oxygen species (ROS) production [86,92,93,97,98,99], oxidative stress injuries, and chronic inflammation resulting from the hyperglycemic environment and ketoacidosis [100].

Previous studies suggest that individuals with diabetes exhibit an early delay in the activation of Th1 cell-mediated immunity, which is a key factor in the ability to resist intracellular infections via the early influx of IFN-producing Th1 cells [101,102].

## 8. Impact of Immunosuppression on the Incidence of Sporotrichosis

The clinical manifestations of the disease vary according to the host immune response, the yeast load, and the depth of inoculum, which ranges from localized skin lesions to cutaneous dissemination or systemic disease; notably, systemic disease has been reported in immunosuppressed individuals in whom the bones, joints, lungs, and CNS were affected [50,103].

**Figure 3:**
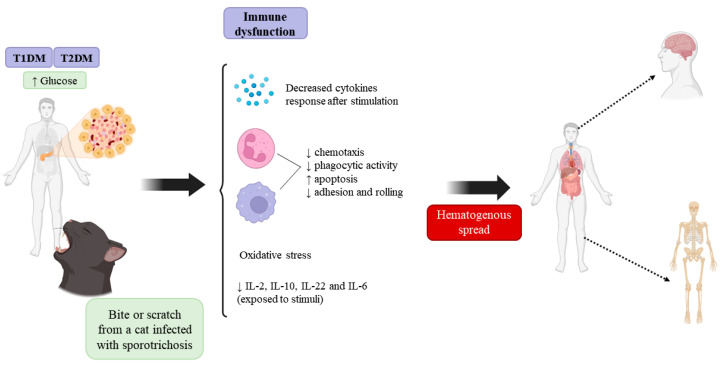
Possible explanations for the greater susceptibility to hematogenous dissemination of *Sporothrix* sp. in individuals with T1DM and T2DM. Individuals with diabetes exhibit immune dysfunction, such as a decreased cytokine response (IL-2, IL-10, IL-6, and IL-22), increased oxidative stress, decreased chemotaxis, phagocytic activity, adhesion and rolling of neutrophils and monocytes/macrophages, and increased macrophage/monocyte and polymorphonuclear cell apoptosis, which allow the fungus to spread via the hematogenous route and reach other organs, such as the heart, kidneys, brain, and bones.

Diabetes itself does not increase the risk of sporotrichosis. However, immunosuppression in individuals with DM can lead to the disseminated form of the disease due to failure to eliminate the pathogen [104] as well as poor infection treatment outcomes [105], as described in Figure 3.

The following are possible explanations for the correlation between patients with diabetes and their greater susceptibility to disseminated cases of sporotrichosis:

(a) Individuals with diabetes have lower levels of NADPH due to high intracellular glucose levels, which impairs the regeneration of molecules that play a fundamental role in antioxidative mechanisms within the cell and consequently increases cell susceptibility to oxidative stress;
(b) A hyperglycemic environment reduces the mobilization of polymorphonuclear leukocytes (PMNs), which alters their adherence, impairs their chemotaxis and phagocytic activity (especially in the presence of acidosis), inhibits the enzymatic activity of glucose-6-phosphate dehydrogenase and the transendothelial transmigration of PMNs, and increases their apoptosis rate [106];
(c) Some microorganisms become more virulent in environments with high glucose contents, in addition to exhibiting greater adhesion to the cells of individuals with diabetes than to the cells of nondiabetic individuals (e.g., *Candida albicans*) due to the inefficient and delayed response of the cellular immune system;
(d) Individuals with diabetes have higher serum levels of IL-1β, IL-6, IL-10, and TNF-α, but they have lower levels of IL-10, IL-22, and IL-6 when exposed to glucose stimulation than control individuals. Therefore, the presence of glucose leads to a greater cytokine production at rest; however, this cytokine production is impaired compared to the absence of glucose [1,107]. Additionally, the production of IL-2, which is important in the inflammatory process and necessary for an effective immune response, decreases after glucose stimulation [108].

The analysis of the treatment period revealed that patients without diabetes were cured more quickly, with an average of 114 days, but individuals with diabetes took approximately 140 days [109] while using oral antifungals, such as itraconazole, terbinafine, and potassium iodide solution.

Previous studies demonstrated the importance of immunological mechanisms in controlling *Sporothrix* sp. infections, as shown by the greater colonization and fungal dissemination in immunosuppressed mouse models [61,62] and immunocompromised patients, such as individuals with HIV [110].

De Beurmann and Ramond (1903) [111] were the first to describe disseminated cutaneous sporotrichosis, which is a rare variant with multiple lesions without extracutaneous involvement [10]. Less than 5% of sporotrichosis cases are disseminated, visceral, or fungal and are most often associated with immunosuppression [112,113,114].

One patient with human immunodeficiency virus (HIV) developed disseminated sporotrichosis in 2012, which led to the development of endocarditis, bilateral endophthalmitis, and lymphatic involvement. After a surgical procedure, isolates of *S. brasiliensis* were obtained from cultures of subcutaneous nodules and mitral valve fragments [115]. In 2020, 10 individuals were diagnosed with oculoglandular syndrome and ophthalmologic lesions caused by sporotrichosis after contact with domestic cats with sporotrichosis [116].

Individuals with diabetes have greater difficulty healing due to hypoxia, inflammation, and oxidative stress [71]. Therefore, these individuals, who were infected by *Sporothrix* spp. subcutaneously via bites and scratches from infected cats, had larger wounds, more secretions, and worse healing than individuals without diabetes.

Although rare, sporotrichosis in the CNS has been described in immunosuppressed patients, primarily in individuals with HIV. One possible reason why sporotrichosis is more likely to lead to meningitis is that high blood glucose levels reduce the ability to eliminate free radicals and compromise the metabolism of various cells, which makes it easier for the fungus to escape the host immune response and reach tissues distant from the inoculation site. The suggestion is that sporotrichosis should be considered a differential diagnosis in cases of chronic meningitis in immunosuppressed patients living in endemic or hyperendemic regions of this disease [110].

According to Freitas et al. [113,114], 14.3% of HIV-infected patients with sporotrichosis present the disseminated form of the disease, with *S. brasiliensis* present in the cerebrospinal fluid (CSF); treatment is challenging due to the difficulty of sterilizing the CSF as well as related complications, such as hydrocephalus [52,117], which results in poor outcomes.

In 2020, a 59-year-old patient who was diagnosed with T2DM and worked at a construction site developed ulcerated rashes on her left ankle and jaw on both sides. She reported that the lesions itched slightly and were sometimes painful but did not report traumatic inoculation. Direct microscopic analysis of exudate smears in 10% potassium hydroxide (KOH) solution or fluorescence staining revealed the presence of slender hyphae. Histopathological examination of the lesions revealed pseudoepitheliomatous hyperplasia in the epidermis, neutrophil abscess in the superficial dermis, and infiltrates of lymphocytes, histiocytes, plasma cells, and some neutrophils in the dermis, with multinucleated giant cells. A fungal culture confirmed the presence of *S. globosa* [49].

Sporotrichosis is currently suggested as a differential diagnosis of chronic meningitis in immunosuppressed patients living in sporotrichosis endemic or hyperendemic areas, and the overall mortality rate is 50% in this context [52,110]. Chronic meningitis caused by *Sporothrix* spp. has been described in patients with immunosuppression due to cirrhosis, organ transplants, diabetes, or Hodgkin’s disease, but sporotrichosis in individuals with HIV is increasingly reported in the state of Rio de Janeiro, Brazil, which may be a result of the sporotrichosis epidemic in cats [52,113,114].

Although cases of chronic meningitis caused by *S. brasiliensis* are still uncommon, the continuous expansion of the sporotrichosis epidemic in Brazil and the exacerbating increase in cases of individuals with diabetes support the importance of additional in-depth studies on *S. brasiliensis*. There have been atypical and more severe cases of sporotrichosis in individuals infected with *S. brasiliensis* [118], even among individuals who are not immunocompromised, which may be due to its greater virulence compared to other species. In murine models, the *S. brasiliensis* strain was the most virulent within the *Sporothrix schenckii* complex and showed dissemination to different organs, including the CNS [12].

CNS involvement in sporotrichosis is a manifestation suggestive of hematogenous dissemination of the fungus resulting from disseminated disease, and it is not always preceded by manifestations of cutaneous sporotrichosis [110,113,114]. According to Louveau et al. [119], another theory of dissemination to the CNS involves the recent discovery that the CNS has a functional lymphatic system that connects with the body’s lymphatic system via lymph nodes located in the cervical region. Therefore, individuals can acquire infections via the respiratory route or traumatic inoculation, which generates a local inflammatory response, and yeast cells are phagocytosed by antigen-presenting cells (APCs), which migrate to the regional lymph nodes via the lymphatic circulation and reach the CNS via this route [18,120].

## 9. Conclusions

The present review elucidated the potential reasons underlying the heightened susceptibility of immunocompromised patients to the hematogenous dissemination of *Sporothrix* sp. and explored the primary alterations resulting from this dissemination. A limited number of studies on the mechanisms by which sporotrichosis disseminates throughout the host’s body and reaches the central nervous system (CNS) exist. However, a variety of factors, such as decreased levels of NADP, reduced mobilization of polymorphonuclear leukocytes, a decreased cytokine response after stimulation, and oxidative stress, may contribute to an increased susceptibility to infection, which underscores the imperative to broaden research efforts to encompass atypical and widespread cases of the disease, particularly among individuals with diabetes mellitus (DM). There is a pressing need to improve early diagnosis and tailor the treatment approaches to ultimately improve patients’ outcomes and survival rates.

## Data Availability

No new data were created or analyzed in this study. Data sharing is not applicable to this article.
